# Looking Forward

**Published:** 1889-11

**Authors:** S. H. Preston


### HALL'S JOURNAL OF HEALTH

TRUTH DEMANDS NO SACRIFICE; ERROR CAN MAKE NONE.

VOL. 36  NOVEMBER, 1889.  No. 11

### LOOKING FORWARD.

BY S. H. PRESTON.

No. 3.

It is strange that a writer so sagacious and scientific as the Dr. has shown himself to be on other subjects should become misled on this matter of population, merely from reading Henry George. It is always a delightful duty to set such a man right with irrefutable facts and figures.

The Dr. seems to take it for granted, or at least would lead the reader to infer, that I am a Malthusian, when the truth is that I thoroughly and totally detest the teachings of Malthus. He was a visionary like Henry George, and a clergyman who, like his profession proverbially, did not himself practice what he preached. As an impediment to increase of population he advocated delay of marriage till late in life so as to insure small or no family, while he himself married soon as he could and had a large family.

The method of Malthus is both undesirable and impracticable. To postpone marriage is to promote prostitution. Marriage is the sole safeguard against profligacy and vice. The more marriages the less immorality. The fact is amply attested in all states of society and conditions of life that marriage not only tends to preserve physical health, but makes men and women happier and better beings. Physicians have come to prescribe early marriage as the preventive or the cure for the errors of youth. It is not well for men and women to live apart after a certain age, and they will not unless compelled by circumstances.

There is no danger that the Malthusian doctrine will ever be adopted to any extent. As far as it might be put in practice it would be found to encourage that cardinal curse of civilization, prostitution. It is mighty poor moral economy to seek to check one evil by substituting a greater. Could we collect the statistics of vice in our large cities we would see the evil effects of deferred marriage, the male profligates being mostly men waiting for more means to provide for a family before taking a wife. Few honorable and pure-minded people will be prevailed upon to procrastinate marriage till passion and the bloom and beauty of the brighter half of life may become the mockery of middle age. And it is best they should not; as otherwise the prudent, provident and principled, the only ones likely too heed the teachings of Malthus and the only people who should propagate, would remain single and childless, leaving the base, the thoughtless and thriftless, the very people who should be restrained to reproduce the race. The general adoption of the Malthusian doctrine would demoralize society and deterioate the human species.

Dr. B. cites as a fact against the statistical increase of population that the descendants of Confucius to-day are but a few thousand, whereas they should number septillions had they doubled at the rapid rate of the whole population of the United States. Deplorable effect of reading Henry George! For no one knowing Dr. Babbitt will believe that such a ridiculous round of reasoning ever originated in his well-balanced brain. Because the posterity of the Chinese Christ, or one family, have not multiplied at the ratio that marks the growth of our remarkable Republic, therefore the most carefully collected statistics are of no account, and the world as a whole enlarges its population very little, if any. The Christian's Christ left no descendants. Fruitful families have utterly died out, dynasties disappeared and the property of peerages reverted to the crown for want of an heir. The maddest of Malthusians never claimed a geometrical growth of every family or every people. While some races, as the Aztecs, have died out, and the Indians are dying out, others breed like rabbits.

There is no universal or uniform rate of propagative productiveness for mankind as a mass. Sardinia once sustained a large population of its own, and at the same time supplied other countries; but it is now stricken with great poverty and its population is very small. On the other hand, Singapore a few years since only supported a small number of savages. It is now a prosperous and populous city. Population follows the fluctuations of the funds for supporting laborers, and is finally fixed by the food supply. So that the rate of population is very variable in different parts of the world, and changes in the same country. The race is like a vast reproductive river, running out of itself and into itself, constantly widening and changing its channels and pouring its overflowing tides into new ones.

The centres of population are steadily shifting. For centuries they have been veering westward. Time was when the Mediterranean was the populous centre of the Eastern world. It is now little else than an idle inland sea locked in a decaying continent. The seat of empire shifts westward with the sun. Effete Europe is dwindling day by day. Whatever of enterprise it yet has is drifting away to the American West. Still, despite its enormous emigration it is gaining greatly in population. The latest complete statistics I have for the entire continent is for the decade from 1870 to 1880, which show an excess of 25,000,000 births over deaths, but estimates that emigration reduced the actual increase to 22,250,000. And this while the increase of population in the United States exceeds the aggregate number of inhabitants in three kingdoms of Europe, viz: Holland, Denmark and Portugal.

England, better conditioned than other countries of continental Europe, is already suffering from a surplus population that supplies one pauper to every thirty-three people, and England has an annual gain equal to the inhabitants of the county of Northampton. Had not its population been pouring out for generations into the "greater Britain" engirdling the globe, the "nook-shotten isle of Albion" would not to day form a foothold for its prolific people.

Statistics show that the Anglo Saxon and Teutonic races are swiftly outstripping all others in population. Compared with them the Latin countries, France, Italy and Spain are fastly falling away as factors in the colonization of humanity. Thus in a little less than a century, from 1788 to 1885, the aggregate populations of these three countries have only increased from 51,000,000 to 82,500,000, while the populations of England and Germany have each trebled during that time. Germany increased from 15,000,000 in 1788 to 45,000,000 in 1885, and England from 12,000,000 to 36,000,000 in the same period; while our States, principally populated by the Anglo-Saxon race, increased from less than 4,000,000 in 1790 to nearly 60,000,000 in 1885, or fifteen times.

The English-speaking peoples of the present hour, those of the United Kingdom and the United States, number one-fifteenth of the race and govern one-third of the globe and one-fourth of its inhabitants. This English race at the beginning of the eighteenth century numbered less than 6,000,000. That number has increased to 100,000,000 to-day. In the period of eighty years the English-speaking people have multiplied five times over, and it is confidently computed that within another century they will outnumber those of all the other civilized countries combined.

M. Kummer, chief of the Bureau of Statistics in Switzerland, has reckoned that the total population of Europe in the year 2000 will be 565,000,000, while Dr. Strong, of New York, reckons that in 1980 the population of Europe will be 534,000,000. Following the same course of computation with the English peoples, and supposing them to increase for a century to come as they did from 1870 to 1880, they would reach the stupendous total of 1,343,000,000.

As stated in a previous article, the most competent compilers of statistics, Behm and Wagner, show the world's increase of population to be 16,778,000 in nineteen months. Now these are facts of statistical tabulation, and no one but Henry George would think of going into septillions with the descendants of Confucius to dispute them. Though they may not have multiplied at the average rate of mankind, it appears that the posterity of other Chinese have so increased that, in spite of their system of infanticide, nine millions died of starvation a few years since, and our government violates its treaty with theirs to prevent them from overwhelming us with their emigrating hordes. Say, Dr., Henry may be trustworthy and all right regarding his single tax theory, but the census seems to upset his Confucian deduction. It should be noted in this connection that the noble, wise and great do not recruit the race with the reckless rapidity of the base, the poor and the ignorant. An instance of the fecundity of the latter class is furnished by the notorious Jukes family. In seventy-five years the twelve hundred criminal and pauper descendants of one vile women up the Hudson cost the State of New York $1,250,000. This without taking into account the sums squandered in their debaucheries and the incalculable entailment of crime and pauperism through succeeding generations. Such is the official record of one family here at home Such folks as the Jukes go far toward making up for the procreating laches of Chinese sages.

In the next number I will tell the Dr. something about Topolobampo Bay.

